# Hip resurfacing arthroplasty at a non-specialist centre

**DOI:** 10.1308/003588414X13824511649850

**Published:** 2014-01

**Authors:** N K Patel, J Wright, S Sabharwal, A Afsharpad, R Bajekal

**Affiliations:** Department of Trauma and Orthopaedic Surgery, Barnet and Chase Farm NHS Trust, Barnet, Hertfordshire,UK

**Keywords:** Metal-on-metal, Hip resurfacing, Arthroplasty, District general hospital, Outcome

## Abstract

**INTRODUCTION:**

Few studies have reported the outcome of hip resurfacing arthroplasty (HRA) with respect to implant characteristics from non-specialist centres. We report the survival, clinical and radiological outcomes of a single surgeon series of HRA with an average follow-up duration of five years.

**METHODS:**

All consecutive HRAs performed by a single surgeon between 2003 and 2011 at a district general hospital were retrospectively examined clinically and radiologically.

**RESULTS:**

A total of 85 patients underwent 109 HRAs (58 male [53.2%] and 51 female patients [46.8%]) with a mean follow-up period of 62 months (range: 12–102 months). The median age was 57 years (range: 25–75 years). The mean acetabular and femoral head component sizes were 54mm (range: 48–64mm) and 48mm (range: 42–58mm) respectively with a mean acetabular inclination angle of 42.9º (range: 20–75º).

The survival rate was 95% with five revisions due to aseptic loosening (*n*=3) and fracture (*n*=2): these were predominantly for female patients (*n*=4), with significantly smaller mean acetabular (51mm, p=0.04) and femoral (44mm, p=0.02) implant sizes. Furthermore, they had a higher mean acetabular inclination angle of 48.1º (*p*=0.74). The mean Oxford hip score was 43.8 (range: 25–48) and the mean University of California Los Angeles (UCLA) activity score was 6.8 (range: 3–10). Radiological findings included heterotopic ossification in 13 (11.9%), radiolucent lines in 6 (5.5%), femoral neck thinning in 2 (1.8%) and femoral neck notching in 5 patients (4.6%).

**CONCLUSIONS:**

We have shown that HRA at a non-specialist centre has short to medium-term outcomes comparable with those at specialist centres. HRA therefore remains a viable option although vigilance is required in case selection and follow-up according to national guidance.

The concept of hip resurfacing with a prosthetic material has existed since the middle of the 20th century, with Smith-Peterson’s use of the Vitallium® (Austenal Laboratories, New York, USA) mould arthroplasty,^1^ followed by Charnley’s work with polytetrafluoroethylene resurfacing.^2^ Charnley’s technique unfortunately tended to fail early, leading to a fall from favour in the technique towards his low friction arthroplasty.^3^

The modern hip resurfacing arthroplasty (HRA) uses a large diameter cobalt-chrome alloy metal-on-metal (MoM) bearing. It was developed in the 1990s by McMinn^4^ for use in young and active patients with osteoarthritis.^5,6^ It has theoretical advantages over conventional total hip replacement (THR) including femoral bone stock preservation, better stability and lower wear characteristics to facilitate high levels of function.^7,8^

Initial outcomes of HRA were similar to THR^9^ with good early-mid term survival,^5,6^ leading to a rapid increase in use and production by various manufacturers. However, there has been much concern regarding long-term follow-up^10^ with significant complications and high failure rates.^5,11^ These include fracture (1–2%),^5^ avascular necrosis and adverse reaction to metal debris (ARMD) in particular.^12–14^ ARMD occurs secondary to metal ion release from edge loading and wear, which depends on implant type, size and position.^15,16^ Women in particular have been shown to have high failure rates, as has the use of the ASR™ prosthesis (DePuy, Warsaw, IN, USA).^17^ These concerns have led to a withdrawal of this particular HRA from the market.

Although these adverse reactions are thought to occur infrequently,^18^ the use of MoM HRA has now become significantly limited to young male patients with isolated primary osteoarthritis and good bone quality.^19,20^ Those patients with HRA in situ require rigorous monitoring according to Medicines and Healthcare products Regulatory Agency (MHRA) guidance.^21^

Nevertheless, the long-term outcomes from specialist centres have been encouraging.^7,18^ Few data from non-specialist centres examining survival and implant characteristics with five-year follow-up are available.^22,23^ We report the outcomes of a single surgeon series of HRA at a UK district general hospital with a five-year follow-up period. The primary endpoint was a need for revision while secondary endpoints were clinical and radiological outcomes with respect to type, size and position of implants.

## Methods

All consecutive patients who underwent HRA by the senior author (RB) between 2003 and 2010 at a typical UK district general hospital were investigated retrospectively. All patients had failed non-operative management with significant complaints of pain, difficulty with activities of daily living and function. The baseline patient demographics are shown in Table 1. All the patients gave informed consent prior to being included in the study. The study was authorised by the local ethics committee and was performed in accordance with the ethical standards of the Declaration of Helsinki.

**Table 1:** Baseline patient demographics

	Patients(*n*=109)
Mean age in years (range)	57 (25–75)
*Sex*	
Male	58 (53.2%)
Female	51 (46.8%)
*Diagnosis* (*hip)*	
Osteoarthritis	97 (89.0%)
Developmental dysplasia	7 (6.4%)
Rheumatoid arthritis	3 (2.8%)
Psoriatic arthritis	2 (1.8%)

### Surgical technique and postoperative management

All procedures were performed using a standard posterior approach, according to the manufacturer’s suggested technique. All acetabular components were press-fit and had a metal backed socket with a metal bearing. Simplex™ antibiotic bone cement (Stryker, Kalamazoo, MI, USA) was used to fix the femoral components. A standardised post-operative regime of care for joint arthroplasty was used. Each patient received 24 hours of prophylactic antibiotic cover with intraoperative calf pumps, thromboembolic deterrent stockings and low molecular weight heparin until discharge as venous thromboprophylaxis. All patients were fully weight bearing with crutches as tolerated following surgery.

### Clinical and radiological evaluation

All patients were followed up clinically and radiologically at 6 weeks, 6 months and 12 months postoperatively with subsequent lifelong annual reviews. Early and late postoperative complications were recorded, including the need for revision, which was the endpoint for survival. Postoperative activity scores were recorded including the Oxford hip score (OHS)^24^ and the University of California Los Angeles (UCLA) activity score^25^ at the latest follow-up visit or by telephone.

Serial radiographic examination included anteroposterior (AP) pelvic and lateral hip radiography to assess for implant position, radiolucent lines (defined as >2mm at the bone implant–cement interface in any of the zones described by Delee and Charnley^26^ for the acetabular component and Amstutz^27^ for the femoral component), fracture and heterotopic ossification (according to Brooker classification, Table 2).^28^

**Table 2:** Brooker classification of heterotopic ossification^28^

Class	Description
I	Islands of bone in the soft tissues
II	Bone spurs arising from proximal femur/pelvis with ≥1cm joint space between ends
III	Bone spurs arising from proximal femur/pelvis with <1cm joint space between ends
IV	Apparent ankylosis of hip joint

Specifically, the acetabular component inclination was measured against the horizontal line between ischial tuberosities, and the femoral component stem shaft angle was defined as the angle between a line along the femoral stem and the proximal femoral canal. Femoral neck width was measured at the implant neck junction on the latest postoperative AP radiography. Thinning was present when there was a reduction of >10% in the same dimension compared with the immediate postoperative radiography.^7^ Femoral neck notching was present when a surgically induced superior or inferior notch of the neck of >1mm was seen on either AP or lateral radiography. These measurements were performed by two authors (NP and JW) using picture archiving and communication system (PACS) software (Kodak, Rochester, NY, USA) with the average used in data analysis.

### Statistical analysis

Statistical analysis was performed using SPSS® version 11.0 (SPSS, Chicago, IL, USA). Mann–Whitney U and Fisher’s exact tests were used for analysis of statistical significance. A *p*-value of ≥0.05 was considered as statistically significant.

## Results

A total of 109 HRA procedures were performed in 85 patients. The mean follow-up duration was 62 months (range: 12–102 months). No patients were lost to follow-up. Of the 109 HRAs, 105 were Birmingham Hip Resurfacing (BHR) (Smith & Nephew, Warwick, UK), 2 were Adept® (DePuy), 1 was Conserve® Plus (Wright, Arlington, TN, USA) and 1 was ASR™ (DePuy). The mean acetabular component size was 54.7mm (range: 48–64mm) and the mean femoral component size was 48.1mm (range: 42–58mm).

**Figure 1:**
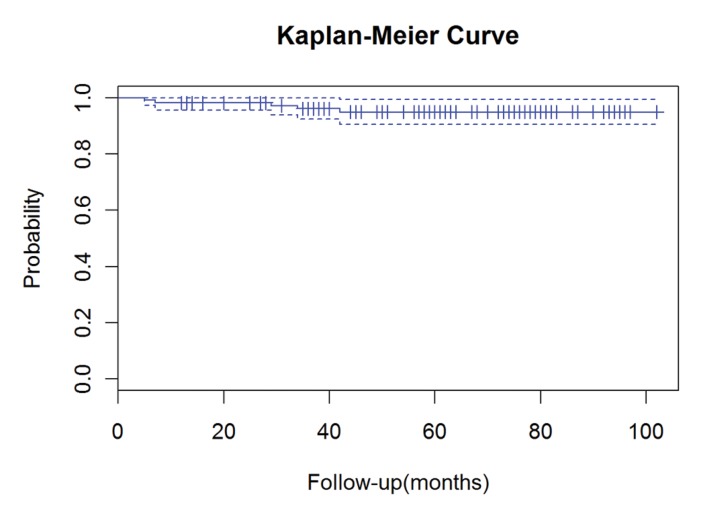
Kaplan–Meier survival curve for all patients with 95% confi dence intervals (dashed lines)

The cumulative survival rate was 95% (95% confidence interval: 90.6–99.3%) and there were five revisions (4.6%). The mean time to revision was 23.4 months (range: 5–42 months). Information regarding the revised patients is shown in Table 3 and a comparison with non-revised patients in Table 4. The Kaplan–Meier survival curve is shown in Figure 1. There were no cases of deep infection.

**Table 3:** Details of patients with revisions

Age / sex	Diagnosis	Implant	Time to revision	Aetiology	Operation	New bearing	Outcome
57 F	OA	BHR	7 mths	Fracture	Stem revision	MoM	Radicular (back) pain
62 F	RA	BHR	29 mths	Aseptic loosening (poor primary fixation)	Cup/stem revision	MoP	Pain free
58 F	OA	ASR™	42 mths	Aseptic loosening (lack of primary fixation)	Cup/stem revision	MoP	Pain free
58 F	DDH	BHR	34 mths	Aseptic loosening (lack of primary fixation)	Cup/stem revision	MoP	Pain free
56 M	OA	BHR	5 mths	Fracture	Stem revision	MoM	Pain free

OA = osteoarthritis; BHR = Birmingham Hip Resurfacing; MoM = metal-on-metal; RA = rheumatoid arthritis; MoP = metal-on-polyethylene; DDH = developmental dysplasia of the hip

**Table 4:** Comparison of patients with and without revisions

	Patients without revision(*n*=104)	Patients with revision(*n*=5)	*p*-value
Mean age (range)	56.9 (25–75)	58.7 (56–62)	0.06
*Sex*			
Male	57 (54.8%)	1 (20.0%)	0.05*
Female	57 (45.2%)	4 (80.0%)	
*Mean implant size*			
Acetabular	54mm(48–64mm)	50mm(48–54mm)	0.04*
Femoral	48mm(42–58mm)	44mm(42–46mm)	0.02*
*Mean inclination angle*			
Acetabular	44.7º(15.9–61.0º)	48.1º(20.8–75.2º)	0.74
Femoral	137.3º(120.1–155.5º)	137.3º(132.4–149.5º)	0.39

Two patients (one male and one female) required revision for a femoral neck fracture, both within one year of surgery. Radiography showed that the female patient had pre-existing femoral neck thinning. The male patient had neither thinning nor notching but the original procedure was performed by a trainee, under supervision. Both patients underwent revision of the femoral components to an uncemented THR, with MoM bearings. At the time of revision there was no evidence of ARMD or osteonecrosis. At the latest follow-up visit, the male patient was symptom free, and the female patient had developed radicular low back pain with a cobalt level of 108nmol/l (acceptable upper limit 119nmol/l) and a chromium level of 69nmol/l (acceptable upper limit 134.5nmol/l).^21^

Three patients required revision for aseptic loosening (two patients acetabular and one patient both acetabular and femoral). The acetabular inclination angles prior to revision were 20º, 60º and 75º respectively. One of these patients had a preoperative diagnosis of developmental dysplasia of the hip and presented with a dislocation secondary to a lack of primary fixation. The other two patients with aseptic loosening were also found to have a lack of primary fixation but no evidence of ARMD or osteonecrosis. All patients had both femoral and acetabular components revised to a metal-on-polyethylene bearing, and are pain free at follow-up. At the time of each revision, samples were sent for microbiological analysis and revealed no growth. Samples sent for histological analysis also did not reveal any specific abnormality.

**Figure 2:**
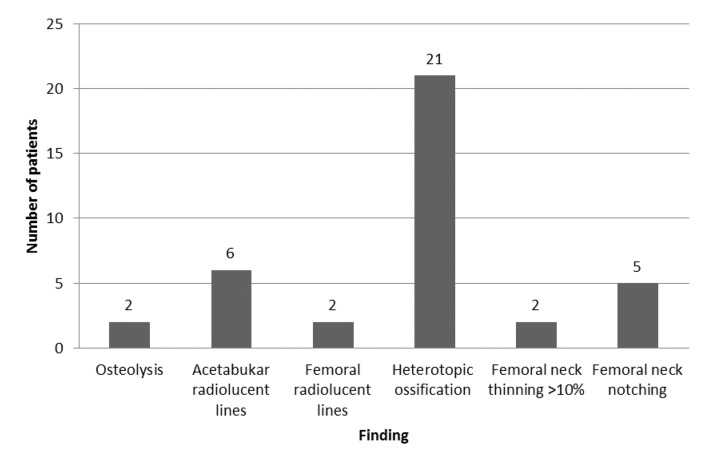
Abnormal radiological fi ndings of all patients

At the latest follow-up visit, the mean OHS was 43.8 (25–48) and the mean UCLA activity score was 6.8 (range: 3–10). Radiological analysis showed that the mean implant angle was 42.9º (range: 20.0–75.0º) and 137.3º (range: 119.3155.5º) for the acetabular (inclination) and femoral (stem shaft angle) components respectively. Neck thinning of >10% was seen in two patients (1.8%). Heterotopic ossification was seen in 21 patients (19.2%): 6 Brooker class I, 5 Brooker class II and 2 Brooker class III. Other radiological findings are shown in Figure 2.

There were four early complications (3.7%) with three surgical site infections (all treated successfully with oral antibiotics) and one deep vein thrombosis (treated with three months of warfarin therapy). There were two patients with unexplained pain (one male, one female) in whom symptoms resolved without any treatment.

## Discussion

We have demonstrated that HRA has a short to mediumterm survival rate of 95% in an independent non-specialist centre despite a relatively high number of female patients. Our patients had good OHS and UCLA activity scores overall. Although age was not an indicator for revision, female sex and smaller implant size were significant risk factors. Interestingly, there were no demonstrated cases of ARMD in our cohort.

Our survival rate is comparable with those for other specialist and non-specialist centres, with rates of 95.899.1% with a mean of 5–6 years of follow-up.^8,22,23^ Six-year results from the Finnish arthroplasty register for hip resurfacing in patients aged <55 years with osteoarthritis between 1991 and 2001 was 94% for each component.^29^ Their study suggested an increased risk of revision in centres with an output of <100 HRAs per year, which is an important consideration for any non-specialist centre performing this procedure. Many of the procedures at our centre were performed in the early period of the study owing to progressively changing referral patterns and increasing vigilance with indications.

The two principal causes of failure in our study were femoral neck fracture and aseptic loosening. Fractures often occur early in the postoperative period,^12^ as was the case in our patients. One suggested cause is osteonecrosis^12^ although there was no macroscopic evidence of this in the two patients in our study. Osteonecrosis can occur from poor surgical technique damaging the extraosseous blood supply to the femoral head^30,31^ or thermal osteonecrosis^32^ associated with bone cement. One patient with a fracture had both femoral neck thinning and notching of the proximal femur. As well as producing a stress riser, notching is also known to increase the risk of osteonecrosis through disruption of the blood supply to the femoral head.^31^ All but one case of notching were noted in the early period of introducing this procedure, demonstrating the steep learning curve associated with HRA.

Rather than loosening *per se*, a lack of primary fixation of the acetabular component was the cause of revision in three patients. This probably occurred from inadequate seating of the acetabular component, thereby hindering bone on-growth on the hydroxyapatite coating. Although radiolucency around the femoral component was not associated with revision in this study, thermal osteonecrosis has been suggested as a cause for this.^32^ Patients undergoing revision THR following HRA do not usually have outcomes comparable with primary THR, particularly for pseudotumours,^33^ yet the patients in our study who had revisions were largely pain free without failure at the latest follow-up visit, which may relate to the absence ARMD.

The risk factors for revision in our study included female sex and smaller implant size. This reproduces the findings of a number of studies including those by McBryde *et al*, who demonstrated through multivariate analysis that once implant size was taken into account, sex no longer influenced the revision rate, reflecting the smaller femoral head sizes in the female population.^20^ Our results were good despite a higher proportion of female patients than the 33–41.2% in other studies.^6,15,16,20^ This may be attributable to use of the BHR dysplasia cup and a lower mean acetabular inclination angle than in other studies (46.2–47º).^6,18,34^ Age was not a risk factor for revision, a finding replicated in both registry data and a prospective series.^17,20^

There were no cases of ARMD (including metallosis, aseptic lymphocytic vasculitis associated lesions or pseudotumours) identified in this study. Conversely, Grammatopolous *et al* found 16 of 53 revisions were for a pseudotumour out of a cohort of 1,375 patients (3.6% revision rate).^33^ This may be explained by our lower patient numbers and that ARMD may be subclinical. Higher acetabular inclination angles have been shown to increase edge loading and wear, causing disruption of fluid film/mixed lubrication, with greater release of metal ions^16^ and possibly ARMD.^34^ Our lack of ARMD may also be explained by the fact that we had a lower mean inclination angle than in other studies, which recommend that 45–55º are safe upper limits for implantation.^16,35^ Although we did not demonstrate that the inclination angle was significantly associated with revision, all three patients with aseptic loosening had angles outside acceptable ranges (20º, 60º and 75º).

Smaller component sizes can be less forgiving with regard to acetabular malpositioning, leading to increased risks of edge loading and impingement.^35^ Measurement of cobalt and chromium levels (with magnetic resonance imaging as appropriate) is now routine in our centre in patients with pain according to the MHRA guidelines.^21^

The vast majority of implants used in this series were BHRs, which may provide some explanation for the good results of this study. Registry data have suggested that ASR™ (DePuy), Conserve® (Wright), Cormet™ (Corin, Cirencester, UK), Durom® (Zimmer, Warsaw, IN, USA) and ReCap® (Biomet, Warsaw, IN, USA) are each associated with a higher revision hazard than the BHR.^17^ Of these, the (*n*ow withdrawn) ASR™ had the greatest hazard ratio. Specifically, the BHR has a ten-year survival rate of 98%^7^ with superior function to and lower revision rates than hybrid THRs.^9^

Heterotopic ossification was particularly common in our patients (19.2%), albeit lower than other published series. Originally described as affecting 21% of THRs,^28^ the rate is often higher in HRAs at 29.5%^36^ and 58.6%.^37^ This relates to greater bone debris from reaming the femoral head and soft tissue stretch compared with conventional THR. Apart from meticulous technique, no other prophylaxis was used routinely for our patients – this may include indomethacin and radiotherapy.^36,37^

### Study limitations

The limitations to this study include the small cohort size although this reflects the typical volume of HRA by a district general hospital surgeon. Pre-operative functional scores were not recorded accurately in our patients, which would have served as a useful baseline. The heterogeneous set of implants makes our results less generalisable although they give an indication of outcome variation. Measuring acetabular component orientation using PACS software may be inaccurate compared with the EBRA (Ein-Bild-Röntgen-Analyse) method.^33^ Measurement of component version on non-standardised cross-table lateral views has been demonstrated to be unreliable.^38^ Computed tomography is the ideal imaging modality for accurate measurement of component version. However, as version only has a weak correlation with adverse wear rates in HRA,^39^ it was not obtained routinely in this patient group. Finally, metal ion levels were not measured routinely as the facilities and funding were not available at that time. Nevertheless, this is now performed in all patients at our centre according to national guidance.^21^

## Conclusions

We have demonstrated that HRA in a non-specialist centre has outcomes similar to those at other specialist centres despite a relatively higher number of female patients. Consequently, HRA still remains a viable option in selected young male patients with osteoarthritis. We have identified predictors of revision, which will help in patient selection. Experienced surgeons should perform these procedures, with good reproducible surgical technique, to avoid component malposition such as high acetabular inclination. There is a small margin for error and a significant risk of complications in comparison with THRs. Although we did not demonstrate any local soft tissue reactions, this remains an important problem, especially in women, who often require smaller implant sizes. We advocate regular lifelong follow-up visits in all HRA patients according to MHRA guidelines.
